# Pulmonary Vasculitides: A Radiological Review Emphasizing Parenchymal HRCT Features

**DOI:** 10.3390/diagnostics11122318

**Published:** 2021-12-09

**Authors:** Stefano Palmucci, Corrado Inì, Salvatore Cosentino, Luigi Fanzone, Stefano Di Pietro, Alessia Di Mari, Federica Galioto, Francesco Tiralongo, Giovanna Vignigni, Stefano Toscano, Gianluca Sambataro, Carlo Vancheri, Giulio Distefano, Antonio Basile

**Affiliations:** 1 Radiology Unit 1, Department of Medical Surgical Sciences and Advanced Technologies “GF Ingrassia”, University Hospital Policlinico “G. Rodolico-San Marco”, University of Catania, 95123 Catania, Italy; corrado.ini@gmail.com (C.I.); salvatorecosentino209@gmail.com (S.C.); luigi.fanzone2@gmail.com (L.F.); stefanodp1608@gmail.com (S.D.P.); alessiadimari@hotmail.com (A.D.M.); federicagalioto91@gmail.com (F.G.); tiralongofrancesco91@hotmail.it (F.T.); stefano.toscano91@gmail.com (S.T.); giuliodistefano@gmail.com (G.D.); basile.antonello73@gmail.com (A.B.); 2 Regional Centre for Interstitial and Rare Lung Disease, Department of Clinical and Molecular Biomedicine, University of Catania, 95123 Catania, Italy; giovannavignigni@gmail.com (G.V.); dottorsambataro@gmail.com (G.S.); vancheri@unict.it (C.V.)

**Keywords:** vasculitis, antibodies, antineutrophil cytoplasmic, multidetector computed tomography, lung diseases, interstitial

## Abstract

Vasculitides represent a heterogeneous group of immune-mediated disorders, characterized by a systemic inflammatory destructive process of the blood vessels resulting either in ischemia or hemorrhage. The organ involved and vessel size influence the pattern of presentation of the pathology. The lung is commonly involved in systemic vasculitides, with heterogeneous clinical, radiological, and histopathological presentations. Primary vasculitides most commonly associated with lung parenchymal involvement include small-vessel antineutrophil cytoplasmic autoantibody (ANCA)-associated vasculitides, such as granulomatosis with polyangiitis (GPA), eosinophilic granulomatosis with polyangiitis (EGPA), and microscopic polyangiitis (MPA). Several studies have reported cases of interstitial lung diseases (ILDs) associated with systemic vasculitis, particularly those positive for ANCA associated vasculitis/vasculitidis: AAV. We have selected from our case series different radiological features of pulmonary vasculitis (i.e., solitary or multiple nodules, cavitary lesions, nodules with centrilobular or peribronchial distribution, airspace consolidations, “crazy paving” appearance, interstitial disease), including cases with interstitial lung alterations. Therefore, the aim of this review is to describe the typical clinical manifestations of vasculitides and their main radiologic features (especially AAV).

## 1. Introduction

As stated in the 2012 Revised International Chapel Hill Consensus Conference (CHCC) Nomenclature of Vasculitides, vasculitis is a systemic inflammatory destructive process of the blood vessels–resulting in ischemic or hemorrhagic alterations. According to the 2012 CHCC Nomenclature, the most widely used approach to classify the primary vasculitis is based on the predominant type of vessels involved: large vessel vasculitis (aorta and its largest branches), medium vessel vasculitis (main visceral arteries), and small vessel vasculitis (capillaries, venules and arterioles)–even if vasculitis of all three major categories may affect arteries of any size [1,2]. Vasculitides can be also broadly divided into primary vasculitis (predominantly idiopathic disorders) or secondary vasculitis, in which the inflammation is related to the presence of a well-defined underlying cause (infections, connective tissue diseases, hypersensitivity or autoimmune disorders). Systemic vasculitides are clinically heterogeneous and should be considered in patients who present with systemic or constitutional symptoms in combination with evidence of single or multi-organ dysfunction. Systemic symptoms are in fact nonspecific, and depending on the organ mainly involved they include fevers, unexplained weight loss, anorexia, weakness and malaise, upper airway disease, emoftoe, serositis ocular inflammation, hematuria and glomerulonephritis, hepatitis, palpable purpura, unexplained ischemic events. Primary pulmonary involvement is a rare disorder, with an incidence of 20–100 cases per million and a prevalence of 150–450 cases per million [3].

The purpose of this paper is to describe main clinical and radiological manifestations of pulmonary vasculitides. Therefore, we have thoroughly reviewed the scientific literature, focusing on anti-neutrophil cytoplasmic autoantibody (ANCA) associated vasculitides, microscopic polyangiitis, granulomatosis with polyangiitis and other rarer forms; for each disease, we have deepened the high-resolution computed tomography (HRCT) patterns, providing radiological features that radiologists should consider to achieve a correct diagnosis.

## 2. Pulmonary Vasculitis

### 2.1. Overview

The overall incidence per annum of primary systemic vasculitis is approximately 20 to 40 cases per million, with an incidence of 20–100 cases per million and a prevalence of 150–450 cases per million. Giant cell arteritis is the most common among primary vasculitides. These diseases may be secondary to other conditions (infectious diseases, connective tissue diseases, malignancies, or autoimmune disorders), or may constitute a primary disorder, in most cases idiopathic, and in which vasculitis represent a primary pathological process [3,4]. The most commonly associated forms with lung involvement include small vessel ANCA–associated vasculitides: granulomatosis with polyangiitis (GPA–formerly Wegener’s granulomatosis), microscopic polyangiitis (MPA), and eosinophilic granulomatosis with polyangiitis (EGPA–also known as Churg–Strauss syndrome). These vasculitides determine a plethora of different clinical, histological and radiological manifestations; moreover, several studies have advocated a correlation between ANCA-associated vasculitis and ILDs [5]. However, primary idiopathic, medium and large-vessel vasculitis, primary immune complex–mediated vasculitis, and secondary vasculitis—may also determine lung involvement [3]. Large-vessel vasculitis predominantly affect the aorta and its largest branches. Pulmonary arteries may be also affected. These vasculitides should be suspected when signs and symptoms of ischemia are present; Takayasu arteritis (TA) and giant-cell arteritis (GCA) are the most common large-vessel vasculitis [6]. 

### 2.2. Radiological Features

Radiological features are extremely variable and often a correct diagnosis may represent a challenge. Chest radiography is often non-specific, and it is hard to define the right pattern of the disease—often underestimating the extension of thoracic involvement. For medium and large-vessel vasculitides, contrast-enhanced computed tomography (CECT) scans and MRI are needed for a correct evaluation of the vessel wall—as well as for a precise evaluation of narrowing/dilation of the lumen. MRI may be preferred in young patients since it avoids the radiation exposure; however, due to the higher spatial resolution and minor presence of artifact, CECT is more accurate than MRI [7]. 

**Figure 1:**
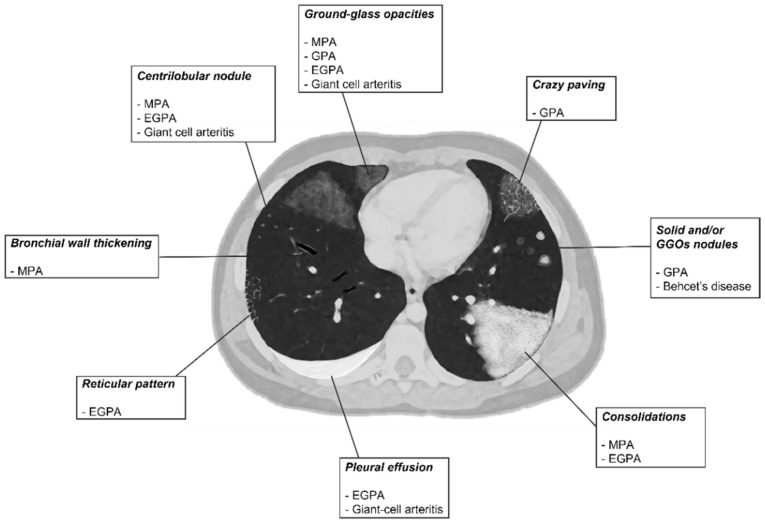
A schematic drawn of parenchymal HRCT features in most common primitive vasculitides whit pulmonary involvement.

HRCT is the most reliable method to assess the distribution, characteristics, and evolution of lung involvement, showing abnormalities even in the absence of clinically significant symptoms (Table 1). Main HRCT findings include vessel wall thickening and aneurysmatic dilatation of pulmonary vessels, scattered multiple uni- or bi-lateral opacities; lung nodules can possibly evolve into cavitary lesion. Micronodules with centrilobular and peribronchial distribution, frequently with no craniocaudal predilection, ground-glass opacities (GGOs) and parenchymal consolidations are often shown. The “crazy paving” pattern is the expression of patchy GGOs associated with smooth septal thickening [6]. Upper airway involvement could be also present. The HRCT pattern of diffuse alveolar hemorrhage associated with vasculitis is characterized by the presence of patchy diffuse ground-glass opacities often associated with areas of consolidation due to the presence of hemorrhagic alveolitis or edema. The distribution is predominantly bilateral, para-hilar, sparing subpleural regions; however, lung volume is usually preserved [8]. A schematic drawn—summarizing all HRCT features of vasculitis described—is proposed in Figure 1.

### 2.3. Microscopic Polyangiitis (MPA)

**Figure 2:**
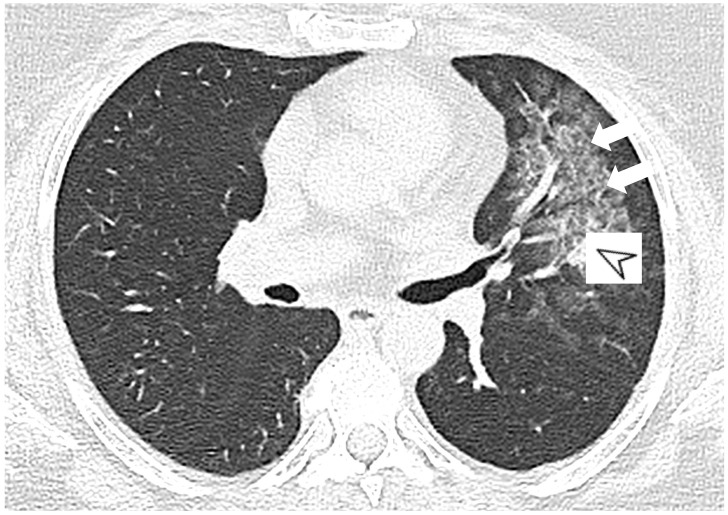
A 52-year-old female with MPA vasculitis. Axial high-resolution CT shows GGO in the left upper lobe (white arrows), due to alverolar hemorrhage; a peribronchial opacity is also depicted in the pulmonary parenchyma (white arrowhead).

**Figure 3:**
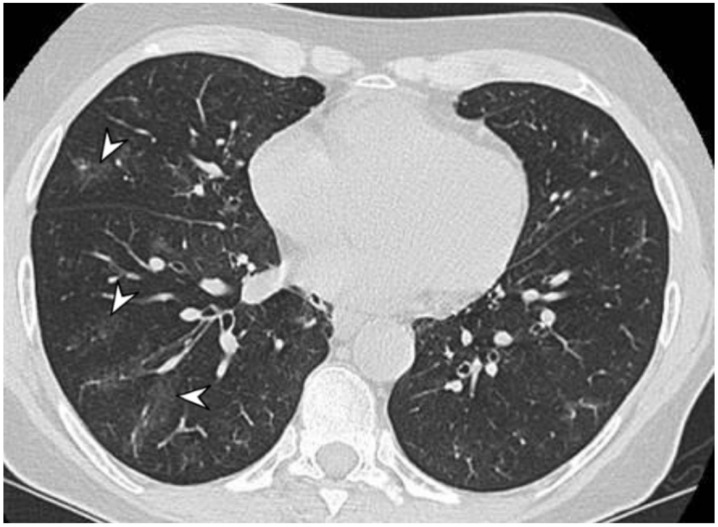
A 57-year-old female with p-ANCA associated vasculitidis. Axial high-resolution CT shows patchy ground-glass opacities (arrowheads), predominantly located in the right lower lobe, sparing subpleural regions.

**Figure 4:**
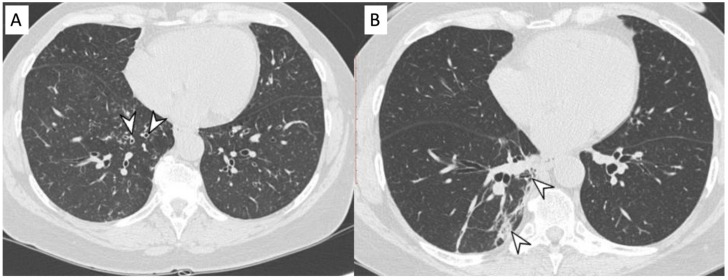
Microscopic Polyangiitis (MPA). Axial CT scan shows micronodules with centrilobular distribution and diffuse bronchial wall thickening (white arrowheads in (**A**)). After treatment, 2 years later, axial CT scan shows the appearance of (focal organizing pattern) focal consolidation (white arrowheads in (**B**)), as clearly depicted in the right lower lobe.

MPA is a clinicopathologic syndrome with pulmonary and renal involvement, characterized by alveolar capillaritis, which leads to alveolar hemorrhage and necrotizing glomerulonephritis. Clinical symptoms are dyspnea and hemoptysis. The HRCT pattern consists of signs of hemorrhagic alveolitis, with focal (Figure 2) or patchy and bilateral GGOs (Figure 3); areas of consolidation and nodules with centrilobular distribution represent further features [9,10]. Other HRCT features are represented by airway lesions, which consist of bronchiolitis, bronchial wall thickening and bronchiectasis (Figure 4) [9].

In MPA, non-granulomatous necrotizing small-vessel vasculitis is the histologic pattern predominant [11]. The diffuse alveolar infiltrates–expression of alveolar hemorrhage–could be depicted not only in MPA, but also in EGPA and Goodpasture’s syndrome [12].

### 2.4. Granulomatosis with Polyangiitis (GPA-Wegener’s Disease)

**Figure 5:**
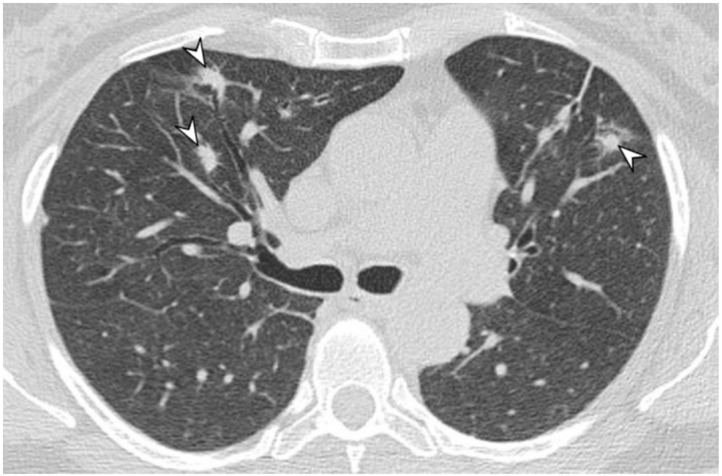
A female patient, with diagnosis of Wegener’s disease. CT image shows multiple bilateral lung nodules (arrowheads).

**Figure 6:**
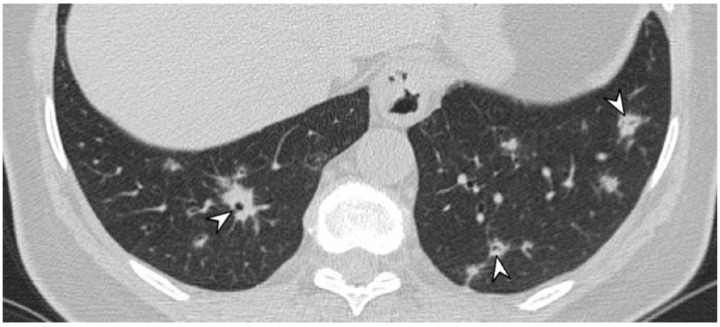
A patient with diagnosis of Wegener’s disease. CT image shows multiple bilateral lung nodules (arrowheads), with air bronchogram sign.

Granulomatosis with polyangiitis (GPA—Wegener’s disease) is a rare disorder with a prevalence of 1–3 cases per 100,000. Symptoms of upper and lower respiratory tract involvement include sinusitis, dyspnea, cough, and hemoptysis. Extrapulmonary manifestations include involvement of kidneys, nervous system (polyneuritis), eyes, skin, muscles, and joints. HRCT abnormalities include solid and/or ground-glass nodules (GGNs) (Figure 5), ranging from few millimeters to several centimeters in size; they often reproduce air bronchogram (Figure 6) and correspond to focal areas of granulomatous inflammation and parenchymal necrosis. In addition, nodular lesions may have a multiple and bilateral distribution along the bronco-vascular bundles—involving mainly the subpleural regions and having no craniocaudal predilection.

**Figure 7:**
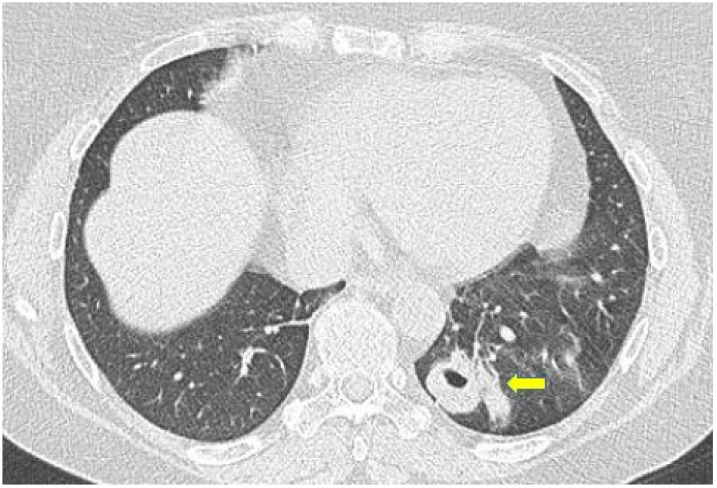
Axial high resolution CT scan in a female patient with Granulomatosis with polyangiitis shows nodule with cavitation presenting thick walls and irregular margins (yellow arrow).

Nodules may cavitate, showing thick walls and irregular margins, and without evidence of calcifications (Figure 7). 

**Figure 8:**
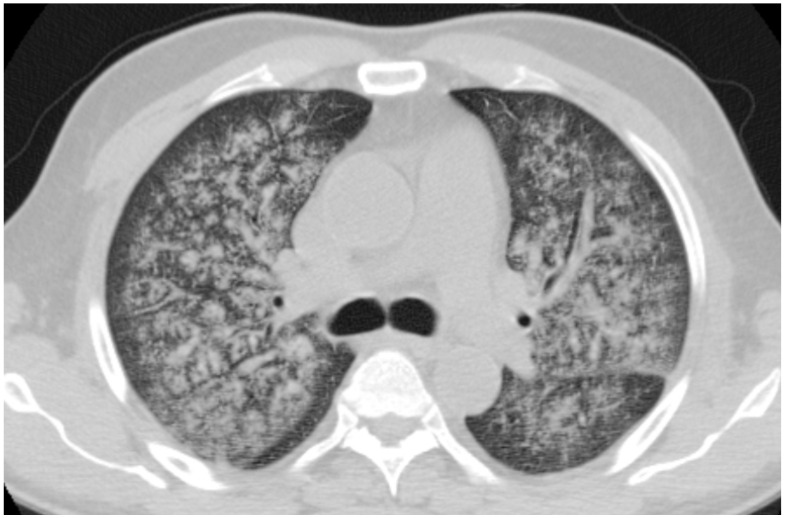
A 64-year-old, male, with fever, weakness, hemoptysis and hematuria. Diagnosis of granulomatosis with polyangiitis. Axial CT shows diffuse hemorrhagic alveolitis diffuse alveolar hemorrhage.

In some cases, HRCT could also reproduce focal “halo sign” with a rim of ground-glass opacity GGO surrounding a pulmonary solid nodule; diffuse alveolar hemorrhage (Figure 8) and crazy paving pattern may be present [13,14,15,16].

GPA shows a histopathological pattern that comprehends areas of pulmonary consolidation with necrosis (in which neutrophilic micro abscesses are often present) and vasculitides; more in detail, arterioles, venules, capillaries may be involved, with focal and eccentric distribution. Capillaritis may be associated with intra-alveolar accumulation of red blood cells. Granulomatous infiltrates consisting of neutrophils, macrophages, lymphocytes, plasma cells, eosinophils, giant cells are implicated. 

### 2.5. Eosinophilic Granulomatosis with Polyangiitis (EGPA-Churg-Strauss)

**Figure 9:**
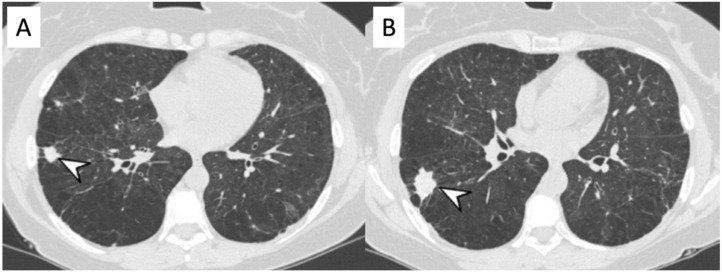
Peripheral nodular consolidations (arrowheads in (**A**,**B**)) located in the right lower lung of a female patient with eosinophilic granulomatosis with polyangiitis. Other pulmonary HRCT abnormalities, represented by emphysematous changes and not related to vasculitis, are also shown.

**Figure 10:**
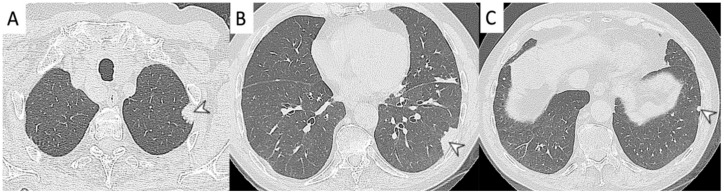
Peripheral consolidations (arrowheads in (**A**–**C**)), located in the left lower lung of a female patient with eosinophilic granulomatosis with polyangiitis.

EGPA or Churg-Strauss syndrome is an allergic granulomatous vasculitis characterized by the following clinical features: a story of asthma and allergic rhinitis which may persist for several years (prodromal period), peripheral blood eosinophilia (second-phase presentation), necrotizing systemic vasculitis (third phase). Clinical manifestations include neurological symptoms (mononeuropathy or polyneuropathy), paranasal sinus abnormalities, coronary arteritis, and lung involvement. Skin involvement with superficial angiitis is also frequent. Purpura, subcutaneous nodules, livedo reticularis, urticarial and necrotic lesions are the most frequent cutaneous findings and reflect leukocytoclastic vasculitis process and eosinophil infiltration. HRCT findings consist of transient and migrant GGOs and consolidations–with bilateral and peripheral locations (Figure 9 and Figure 10); airway alterations are typically found, with diffuse and irregular bronchial wall thickening and bronchial dilatation; in some cases, inhomogeneous lung attenuation could be depicted, due to the obstruction of small airways [17,18,19]. Other characteristics include small nodules with peribronchial and centrilobular distribution, regular and smooth interlobular septal thickening, and pleural effusion. EGPA is characterized by a prominent eosinophil-rich inflammatory infiltrate. More in detail, pulmonary infiltrates may have a random distribution, and they could be migratory. Necrotizing vasculitis could be present. 

### 2.6. Takayasu Arteritis (TA)

**Figure 11:**
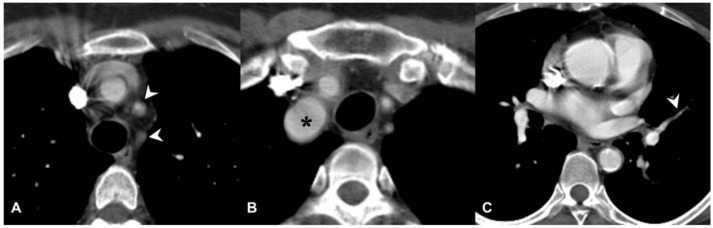
Wall thickening in a patient with Takayasu arteritis. Occlusions of left subclavian artery and common carotid artery (white arrowheads) are depicted in the figure (**A**); dilation of brachiocephalic trunk is also evident in (**B**) (black asterisk). Stenosis of pulmonary segmental artery could be also appreciated in figure (**C**) (white arrowhead), due to granulomatous inflammation of the arterial wall.

TA is an idiopathic chronic arteritis of a large artery, usually resulting in stenosis; it predominantly affects the aorta and/or its major branches. Onset usually occurs before the age of 50 years. The disease is more common in Asia, but it could be found worldwide; it usually affects young women. The estimated annual incidence of TA is 0.12–0.26 cases/100,000 [5]. Pulmonary artery involvement occurs in 50–80% of patients and represents often a late manifestation. TA is characterized by granulomatous inflammation of the arterial wall with intimal proliferation and fibrosis of the media and adventitia, which leads to wall thickening (Figure 11), stenosis, occlusion, and, less frequently, post-stenotic dilation and aneurysm formation [5]. 

Since clinical and laboratory findings are typically nonspecific, imaging findings are necessary to achieve an accurate diagnosis of TA. Takayasu arteritis should be detected during active phase of pathology and before arterial stenosis, when the disease is still reversible. The most typical radiological features are represented by stenosis or occlusion, involving the segmental and subsegmental arteries and less commonly lobar or main pulmonary arteries [20]. CT manifestations of pulmonary artery involvement include wall thickening and enhancement in early phases, whereas mural calcium deposition, and luminal stenosis or occlusion are depicted in the chronic phases. MRI and CT may show contrast enhancement of the thickened walls—when activity is present [5]. Occasionally, pulmonary-systemic shunts have been described in late-phase of TA. Pulmonary artery occlusion could occur in late-phase TA; thus, when a pulmonary artery occlusion of unknown origin is found, late-phase TA should always be considerate [21]. Fluorine-fluorodeoxyglucose (18F-FDG) positron emission tomography (PET) is a useful indicator of the inflammation activity of the vessel walls.

### 2.7. Giant-Cell Arteritis (GCA-Temporal Arteritis)

GCA is the most common vasculitis of large- and medium-sized arteries. It affects almost exclusively individuals >50 years of age. In this age-group, the estimated prevalence of GCA is 278 per 100,000 persons in the United States and is even higher in northern Europe [5]. GCA predominantly affects the extracranial carotid branches and the aorta; main CT and MRI findings are similar to those for TA, with arterial wall thickening, stenosis and aneurysm. Aortic GCA usually remains asymptomatic during the early phase and may cause serious complications in the late phase, such as aneurysms and dissections. Differently to arteriosclerosis, aneurysms in GCA more frequently involve the thoracic aorta and seem more prone to dissection; involvement of the lungs is considered rare in this vasculitis, and may be characterized by nodules, infiltrates, lymphocytic alveolitis, alveolar hemorrhage, and unilateral pleural effusion [22,23]. As in TA, 18F-FDG PET is useful in the demonstration of active disease and in follow-up; however, it must be considered that the interpretation is more difficult than in TA because GCA patients are usually older and there is often concomitant arteriosclerosis [5].

### 2.8. Variable Vessel Vasculitis (VVV)

VVV is a class of vasculitis in which it cannot be identified a type of vessel predominantly affected. Vessels of any size (small, medium, and large) and type (arteries, veins, and capillaries) could be involved [2]. Behcet’s disease—a rare chronic multisystemic disorder—is included in this group of vasculitis. 

It typically presents with eyes involvement (uveitis), recurrent oral and genital ulcers. It may also affect other organs and systems, such as joints, the gastrointestinal system, central nervous system, cardiovascular system, and the lungs [23,24]. It usually presents in the second or third decade of life and the thoracic involvement is seen in 1–8% of cases. Parenchymal lesions commonly associated with Behcet’s disease are subpleural alveolar infiltrates and wedge-shaped or ill-defined rounded areas with increased opacity; these pulmonary alterations represent focal vasculitis process, areas of infarction, hemorrhage or focal atelectasis [23]. Hemoptysis is the most common presenting symptom and is one of the leading causes of death. Vascular involvement is the most common cause of mortality. Venous involvement (85% of the vascular involvement) is more frequent than arterial involvement [8]. The pulmonary arteries are the second most common site of arterial involvement, behind the abdominal aorta. Behçet disease is the most common cause of pulmonary artery aneurysm; due to inflammation of the vasa vasorum of the tunica media with consequent destruction of the elastic fibers and dilation of the vessel lumen. However, immunosuppressant treatment determines complete resolution of aneurysms in up to 75% of patients. Thickening of the vessel wall can be observed in the aorta and superior vena cava. Superior vena cava thrombosis, often associated with thrombosis of other mediastinal veins, is relatively common [5]. 

### 2.9. Vasculitis Associated with Probable Aetiology

Some infectious agents have a role in the onset of vasculitis and in the revised Chapel Hill Nomenclature we can find—in the subgroup “vasculitis-associated with probable aetiology”—three infections associated vasculitis: HCV-related cryoglubulinaemic vasculitis, HBV-polyarteritis nodosa (PAN) and syphilis-associated vasculitis. The most frequent virus associated with vasculitis is HCV cryoglobulinemic vasculitis. Other infectious agents are the cause of some vasculitis such as human immunodeficiency virus (HIV), parvovirus and EBV. 

**Figure 12:**
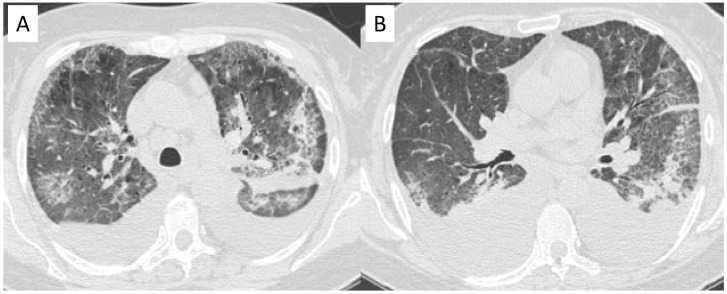
Cryoglobulinemic vasculitis in a patient with chronic renal failure and dyspnoea. Axial HRCT images show diffuse areas of ground-glass attenuation, due to alveolar hemorrhage, with interlobular and intralobular septal thickening. Pleural effusions are also shown in the figures (**A**,**B**).

Patients with vasculitis could develop infections as a consequence of their treatment: immunosuppressant, steroids and immunomodulating agents. Infections occur frequently and represent one of the major causes of death in vasculitis, observed especially in elderly patients and in cases of poor general conditions. In cases with higher risk to develop infections it is necessary to check the immunological status of the patients and improve therapeutic strategies [19]. Cryoglobulinemic vasculitis is a rare condition in patients with serum cryoglobulins and it usually consist of bilateral lung infiltrates and signs of alveolar hemorrhage (Figure 12) [25]. Lung involvement is rarer than in other vasculitides—even if pulmonary alterations have been described. 

### 2.10. ILD in ANCA-Associated Vasculitides

#### 2.10.1. Epidemiology

These small blood vessels vasculitides—as previously described–are characterized by ANCA autoantibodies-which are specific for cellular antigens located in the cytoplasmic granules of neutrophils or in the lysosomes of monocytes. The two main ANCA auto-antigens involved in the pathogenesis of vasculitis are myeloperoxidase (MPO-ANCA) and proteinase 3 (PR3-ANCA). PR3-ANCA is mostly associated with GPA, while MPO-ANCA is essentially found in MPA [26]. Interstitial lung alterations are reported in patients with diagnosis of vasculitis, with a slight predominance of men (60–65%) [16,27]; the age at presentation is older than 65 years, and seems to be much higher in MPA patients [16,27]. Fibrotic and interstitial alterations have been reported with different percentage values: about 23% in GPA, and 2.7–45% in MPA patients. From an epidemiological point of view, the association between AAV and ILD is more frequently in Japanese patients than Western patients: the reason could be the higher prevalence of MPO-ANCA, the increased frequency of pulmonary alterations and the development of diffuse alveolar hemorrhage in these patients [9]. Pulmonary interstitial involvement in AAV have been associated with unfavorable outcomes [16].

#### 2.10.2. Morphological Patterns and Imaging

The onset of ILD is usually concomitant to the diagnosis of AAV (36–67% of patients); however, in some cases ILD may precede the diagnosis (14–85 %) or develops afterwards (8–21%); different authors in literature have reported and analyzed the time of onset of ILDs in patients with pulmonary vasculitis (Table 2) [16].

In MPA patients, HRCT scans show lung abnormalities, and very often reproduce ILD patterns. A Japanese study on a group of 150 patients reported pulmonary alterations in 97% of patients; these included interstitial lung lesions (66%), airway lesions (66%), pleural abnormalities and emphysematous lesions. Therefore, interstitial involvement in ANCA-vasculitis could be frequently depicted, with several fibrosing patterns which present a symmetrical distribution in 50–100% of patients, affecting predominantly lower lobes and the peripheral lung regions [28,29].

**Figure 13:**
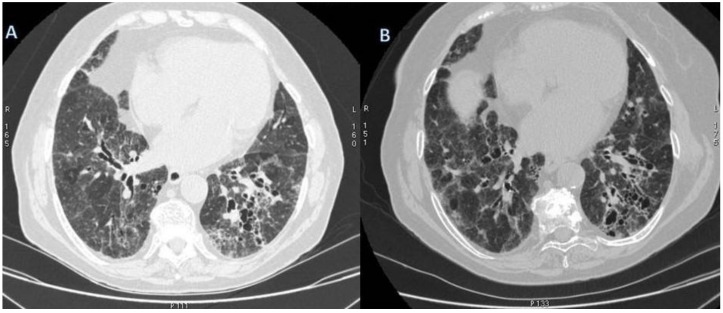
A 76-year-old female patient with GPA. Axial high-resolution CT shows irregular bronchial dilatation and interstitial lung alterations with interlobular and intralobular septal thickening and reticulations, predominantly located in the lower lobe (**A**). After six-months follow-up interstitial and bronchial are still recognizable (**B**).

**Figure 14:**
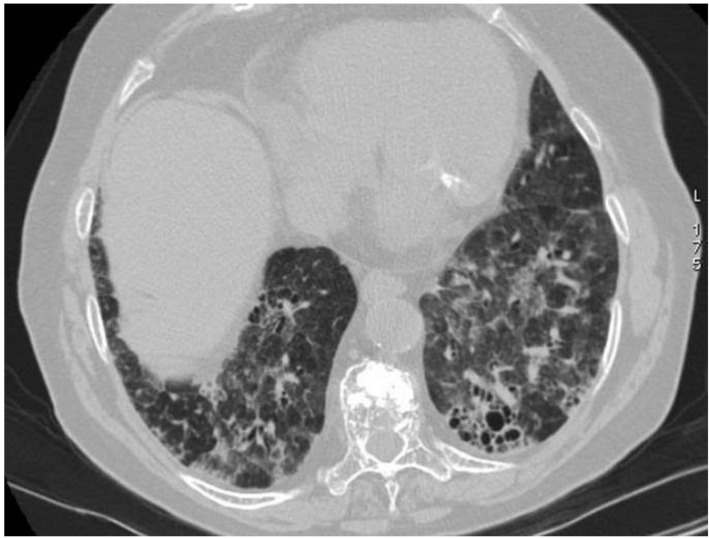
CT scan shows subpleural reticulation and architectural distortion in the lower lobes in an 81-year-old female with AAV. Small, rounded cysts with thick walls are distributed in concentric layers in the subpleural region of the lower left lobe (honeycombing).

**Figure 15:**
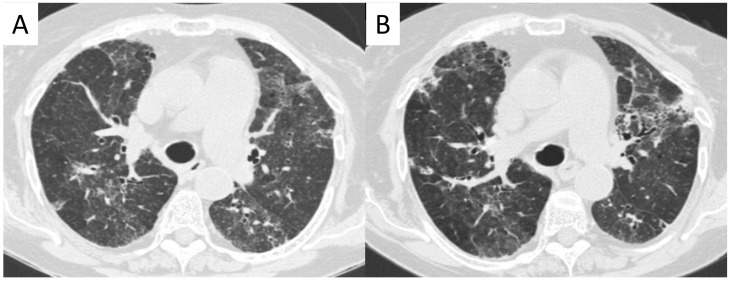
An 80-year-old female patient with granulomatosis with polyangiitis (GPA). HRCT shows irregular bronchial wall thickening, bronchial dilatation, and interstitial lung alterations with interlobular septal thickening and GGOs. It is possibly to appreciate areas of different attenuation of lung parenchyma, resembling the appearance of the so called “mosaic attenuation” (**A**). Follow-up CT (**B**), acquired one year later, confirms the presence of these alterations.

Honeycombing areas, which are due to irreversible interstitial fibrosis, remain unchanged during follow-up studies (Figure 13), while reversible lung alterations may improve [30]. Usual interstitial pneumonia (UIP) and nonspecific interstitial pneumonia (NSIP) are the most frequent interstitial patterns found in chest HRCT of patients with AAV. UIP represents the main frequent pattern (50–57%), followed by NSIP (7–31%) and desquamative interstitial pneumonia (DIP) (14%) [33,34]. Namely, UIP is the mainly common pattern associated with AAV. HRCT shows the presence of honeycombing with basal and subpleural predominance, GGOs, nodules, consolidation, cysts, interlobular septal thickening and airway alterations consisting mainly of peripheral traction bronchiectasis (Figure 14 and Figure 15).

NSIP is characterized by the presence of symmetric and bilateral GGOs, predominantly located in the lower lobe—superimposed on areas of interlobular and intralobular septal thickening, reticulations; traction bronchiectasis could be also depicted. In a retrospective study of 12 cases, a French group has described UIP pattern in 6 cases and NSIP in one case; unspecified interstitial diffuse pneumonia was reported in 5 cases [33,34]. Foulon et al. described 17 patients with pulmonary fibrosis and AAV—showing honeycombing, reticular intralobular opacities and traction bronchiectasis in all the patients, and GGOs in some patients [27,34]. However, the association between fibrosis and AAV have been reported only in small retrospective studies or case reports; in these published reports, the specific characteristics were elder age, male predominance, presence of MPO ANCA. Prognosis is based on pathologic and radiological pattern.

In a cohort of 49 cases with pulmonary fibrosis associated with AAV, 18 patients have shown UIP pattern on HRCT examination [26]. Homma et al.—in a study of 31 patients—have demonstrated a radiological association between MPO-ANCA associated vasculitis and pulmonary fibrosis, showing the presence of reticulonodular shadows, honeycombing and decreased lung volume—predominantly located in the lower lobes and peripheral lung regions [35]. Ando et al. analyzed 61 patients with idiopathic pulmonary fibrosis through HRCT scans—detecting that nine MPO-ANCA-positive patients showed traction bronchiectasis, honeycombing and subpleural reticular alterations [36]. Recent studies also have described an interesting association between ILD, in the form of pleuroparenchymal fibroelastosis (PPFE), and ANCA-vasculitis, characterized by a predominant and progressive fibrotic alteration involving the sub pleural regions and parenchymal areas of upper lung lobes [31,37].

Since idiopathic pulmonary fibrosis represent the most common type of idiopathic ILD, the progressive fibrosing ILD form represents a phenotypic sunset of interstitial lung disease characterized by progressive fibrosis of the lung. The treatment of progressive fibrosing ILD (PF-ILD) includes antifibrotic drugs such as nintedanib and pirfenidone. This treatment seems to improve survival in patients with fibrosing evolution of the disease and slow progression of lung fibrosis. Furthermore, in patients with ILD and AAV, some lung alterations, such as GGO, consolidations and interlobular septal thickening may resolve, partially or completely, after proper therapy.

The consensus statement (American Thoracic Society (ATS) and European Respiratory Society (ERS) 2002) on the classification of the ILD described unclassifiable ILD as an interstitial idiopathic lung disease that cannot be categorized based on clinical, radiological and/or pathological findings. Several studies reporting that ILD remains unclassifiable in 10% of all ILD patients and the prognosis is intermediate between fibrosing and non-fibrosing ILD [16,31,38,39].

## 3. Conclusions

The HRCT pattern–mainly nodules, GGO, consolidations, fibrotic changes-of pulmonary vasculitides shows various and often not pathognomonic alterations. The knowledge of main HRCT findings—related to specific clinical, laboratory and pathological data—should be mandatory for the assessment of early and correct diagnosis of pulmonary vasculitis. However, the diagnosis is often delayed—since that other disease could have similar clinical and radiological manifestations. A multidisciplinary approach is strongly recommended, in order to combine clinical features and radiological and morphological data, and to provide a correct treatment.

## Figures and Tables

**Table 1:** HRCT features of most common primitive vasculitides with pulmonary involvement; GGOs: ground-glass opacities; C.E.: contrast enhancement [6].

Vasculitis	HRCT Features
MPA	GGOs due to hemorrhagic alveolitis (common); consolidation, nodules with centrilobular distribution (less common)
GPA	Solid nodules, GGOs due to hemorrhagic alveolitis (common); halo sign, crazy paving (less common)
EGPA	Migrant GGOs, transient consolidation, irregular bronchial wall thickening, small nodules with peribronchial and centrilobular distribution, pleural effusion.
Takayasu arteritis	Stenosis and/or occlusion of segmental arteries; stenosis and/or occlusion of lobular or main pulmonary arteries (less common); C.E. of vessel wall may be evident
Giant-cell arteritis	Aneurism or dissection of the thoracic aorta (common); nodules, GGOs, monolateral or bilateral pleural effusion (less common)
Bechet’s disease	Subpleural alveolar infiltrates and wedge-shaped or ill-defined rounded areas with increased opacity, pulmonary artery aneurism

**Table 2:** The association of AAV and ILDs according to the literature (PF = pulmonary fibrosis).

Author	Ref	Patients	Onset of ILD
Comarmond et al., 2014	[26]	49 ANCA and PF: Typical UIP 43% Atypical UIP 14% Fibrotic NSIP 7% NSIP 9,5%	Preceding 45% Concomitant 43% Posterior 12%
Foulon et al., 2008	[27]	17 ANCA and ILD: Probable PF 70% UIP 17%	Preceding 76% Concomitant 24%
Arulkumaran et al., 2011	[28]	194 MPA-14 MPA and ILD: Idiopathic pulmonary fibrosis: 57% DIP: 14% NSIP: 7%	Preceding 14% Concomitant 64% Posterior 21%
Tzelepis et al., 2010	[29]	33 MPA-13 MPA with PF: UIP 54% NSIP 31%	Preceding 53%
Casares et al., 2015	[30]	28 MPA-9 MPA with PF: UIP 66% UIP probable 22%	Preceding 55% Concomitant 45%
Huang et al., 2014	[31]	67 MPA-19 MPA with PF: UIP: 28%	Preceding 68% Concomitant 32%
Kagiyama et al., 2015	[32]	504 ILD-36 ANCA with ILD	Preceding 100%

## References

[1] Jennette J.C., Falk R.J., Andrassy K., Bacon A.P., Churg J., Wolfgang L.G., Christiaan Hagen E., Hoffman G.S., Hunder G.G., Kallenberg C.G.M. (1994). Nomenclature of systemic vasculitides: The proposal of an international consensus conference. Arthritis Rheum..

[2] Jennette J.C., Falk R.J., Bacon A.P., Basu N., Cid M.C., Ferrario F., Flores-Suarez L.F., Gross W.L., Guillevin L., Hagen E.C. (2013). 2012 revised International Chapel Hill Consensus Conference nomenclature of vasculitides. Arthritis Rheum..

[3] Brown K.K. (2006). Pulmonary vasculitis. Proc. Am. Thorac. Soc..

[4] Maffessanti M., Dalpiaz G. (2004). Diffuse Lung Disease—Clinical Features, Pathology, HRCT.

[5] Alba M.A., Flores-Suárez L.F., Henderson A.G., Xiao H., Hu P., Nachman P.H., Falk R.J., Jennetteet J.C. (2017). Interstitial lung disease in ANCA vasculitis. Autoimmun. Rev..

[6] Castañer E., Alguersuari A., Andreu M., Gallardo X., Spinu C., Mata J.M. (2012). Imaging findings in pulmonary vasculitis. Semin. Ultrasound CT MR.

[7] Nasser M., Cottin V. (2018). Alveolar hemorrhage in vasculitis (primary and secondary). Semin. Respir. Crit. Care Med..

[8] Ceylan N., Bayraktaroglu S., Erturk S.M., Savas R., Alper H. (2010). Pulmonary and vascular manifestations of Behcet disease: Imaging findings. AJR Am. J. Roentgenol..

[9] Yamagata M., Ikeda K., Tsushima K., Iesato K., Abe M., Ito T., Kashiwakuma D., Kagami S., Iwamoto I., Nakagomi D. (2016). Prevalence and responsiveness to treatment of lung abnormalities on chest computed tomography in patients with microscopic polyangiitis: A multicenter, longitudinal, retrospective study of one hundred fifty consecutive hospital-based Japanese patients. Arthritis Rheumatol..

[10] Casal A., Pereiro T., Valdés L. (2018). Pulmonary vasculitis: An update. Arch. Bronconeumol..

[11] Jennet J.C., Falk R.J. (1997). Small-vessel vasculitis. N. Engl. J. Med..

[12] Schnabel A., Reuter M., Csernok E., Richter C., Gross W.L. (1999). Subclinical alveolar bleeding in pulmonary vasculitides: Correlation with indices of disease activity. Eur. Respir. J..

[13] Chung M.P., Yi C.A., Lee H.Y., Han J., Lee K.S. (2010). Imaging of pulmonary vasculitis. Radiology.

[14] Gaudin P.B., Askin F.B., Falk R.J., Jennette J.C. (1995). The pathologic spectrum of pulmonary lesions in patients with anti-neutrophil cytoplasmic autoantibodies specific for antiproteinase 3 and anti-myeloperoxidase. Am. J. Clin. Pathol..

[15] Mahmoud S., Ghosh S., Farver C., Lempel J., Azok J., Renapurkar R.D. (2016). Pulmonary vasculitis: Spectrum of imaging appearances. Radiol. Clin. N. Am..

[16] Alba M.A., Jennette J.C., Falk R.J. (2018). Pathogenesis of ANCA-associated pulmonary vasculitis. Semin. Respir. Crit. Care Med..

[17] Katzenstein A.L. (2000). Diagnostic features and differential diagnosis of Churg-Strauss syndrome in the lung: A review. Am. J. Clin. Pathol..

[18] Von Vietinghoff S. (2016). Pulmonary manifestations of vasculitis. Pneumologie.

[19] Guillevin L. (2013). Infections in vasculitis. Best Pract. Res. Clin. Rheumatol..

[20] Matsunaga N., Hayashi K., Sakamoto I., Ogawa Y., Matsumoto T. (1997). Takayasu arteritis: Protean radiologic manifestations and diagnosis. Radiographics.

[21] Travis W., Colby T., Lombard C., Carpenter H. (1990). A clinicopathologic study of 34 cases of diffuse pulmonary hemorrhage with lung biopsy confirmation. Am. J. Surg. Pathol..

[22] Anaev E.K., Baranova I.A., Belevsky A.S. (2018). Pulmonary vasculitis: Diagnosis and treatment. Ter. Arkhiv.

[23] Adams T.N., Zhang D., Batra K., Fitzgerald J.E. (2018). Pulmonary manifestations of large, medium, and variable vessel vasculitis. Respir. Med..

[24] Erkan F., Gül A., Tasali E. (2001). Pulmonary manifestations of Behçet’s disease. Thorax.

[25] Feragalli B., Mantini C., Sperandeo M., Galluzzo M., Belcaro G., Tartaro A., Cotroneo A.R. (2016). The lung in systemic vasculitis: Radiological patterns and differential diagnosis. Br. J. Radiol..

[26] Comarmond C., Crestani B., Tazi A., Hervier B., Adam-Marchand S., Nunes H., Cohen-Aubart F., Wislez M., Cadranel J., Housset B. (2014). Pulmonary fibrosis in antineutrophil cytoplasmic antibodies (ANCA)-associated vasculitis: A series of 49 patients and review of the literature. Medicine.

[27] Foulon G., Delaval P., Valeyre D., Wallaert B., Debray M.P., Brauner M., Nicaise P., Cadranel J., Cottin V., Tazi A. (2008). ANCA-associated lung fibrosis: Analysis of 17 patients. Respir. Med..

[28] Arulkumaran N., Periselneris N., Gaskin G., Strickland N., Ind P.W., Pusey C.D., Salamae A.D. (2011). Interstitial lung disease and ANCA-associated vasculitis: A retrospective observational cohort study. Rheumatology.

[29] Tzelepis G.E., Kokosi M., Tzioufas A., Toya S.P., Boki K.A., Zormpala A., Moutsopoulos H.M. (2010). Prevalence and outcome of pulmonary fibrosis in microscopic polyangiitis. Eur. Respir. J..

[30] Casares F.M., Gonzalez A., Fielli M., Caputo F., Bottinelli Y., Zamboni M. (2015). Microscopic polyangiitis associated with pulmonary fibrosis. Clin. Rheumatol..

[31] Huang H., Wang Y.X., Jiang C.G., Liu J., Li J., Xu K., Xu Z.J. (2014). A retrospective study of microscopic polyangiitis patients presenting with pulmonary fibrosis in China. BMC Pulm. Med..

[32] Kagiyama N., Takayanagi N., Kanauchi T., Ishiguro T., Yanagisawa T., Sugita Y. (2015). Antineutrophil cytoplasmic antibody-positive conversion and microscopic paolyangiitis development in patients with idiopathic pulmonary fibrosis. BMJ Open Respir. Res..

[33] Hervier B., Pagnoux C., Agard C., Haroche J., Amoura Z., Guillevin L., Hamidou M.A., French Vasculitis Study Group (2009). Pulmonary fibrosis associated with ANCA-positive vasculitides. Retrospective study of 12 cases and review of the literature. Ann. Rheum. Dis..

[34] Katsumata Y., Kawaguchi Y., Yamanaka H. (2015). Interstitial lung disease with ANCA-associated Vasculitis. Clin. Med. Insights Circ. Respir. Pulm. Med..

[35] Homma S., Matsushita H., Nakata K. (2004). Pulmonary fibrosis in myeloperoxidase antineutrophil cytoplasmic antibody-associated vasculitides. Respirology.

[36] Ando Y., Okada F., Matsumoto S., Mori H. (2004). Thoracic manifestation of myeloperoxidase-antineutrophil cytoplasmic antibody (MPO-ANCA)-related disease. CT findings in 51 patients. J. Comput. Assist. Tomogr..

[37] Bargagli E., Conticini E., Mazzei M.A., Cameli P., Guerrini S., D’Alessandro M., Frediani B., Pleuroparenchymal Fibroelastosis (PPFE) Siena Unit, Italy (2021). Pleuroparenchymal fibroelastosis in interstitial lung disease with antineutrophil cytoplasmic antibody-associated vasculitis. Clin. Exp. Rheumatol..

[38] Shumar J.N., Chandel A., King C.S. (2021). Antifibrotic therapies and progressive fibrosing interstitial lung disease (PF-ILD): Building on INBUILD. J. Clin. Med..

[39] Skolnik K., Ryerson C.J. (2016). Unclassifiable interstitial lung disease: A review. Respirology.

